# Effects of the Positive Allosteric Modulator of Metabotropic Glutamate Receptor 5, VU-29, on Impairment of Novel Object Recognition Induced by Acute Ethanol and Ethanol Withdrawal in Rats

**DOI:** 10.1007/s12640-017-9857-z

**Published:** 2018-01-02

**Authors:** Marta Marszalek-Grabska, Ewa Gibula-Bruzda, Anna Bodzon-Kulakowska, Piotr Suder, Kinga Gawel, Joanna Filarowska, Joanna Listos, Wojciech Danysz, Jolanta H. Kotlinska

**Affiliations:** 1 Department of Pharmacology and Pharmacodynamics, Medical University, Chodzki 4A, 20-093 Lublin, Poland; 2 Department of Biochemistry and Neurobiology, AGH University of Science and Technology, Krakow, Poland; 3 Department of Experimental and Clinical Pharmacology, Medical University, Lublin, Poland; 4 Merz Pharmaceuticals GmbH, Frankfurt am Main, Germany

**Keywords:** Ethanol, VU-29, mGlu5 receptor, Novel object recognition

## Abstract

Glutamate is essential for learning and memory processes, and acute and chronic exposures to ethanol (or protracted abstinence) alter glutamatergic transmission. In the current study, we investigated the effects of VU-29, positive allosteric modulator of metabotropic glutamate 5 (mGlu5) receptor, on the acute ethanol- and ethanol withdrawal-induced impairment of novel object recognition (NOR) task in rats. The influence of VU-29 (30 mg/kg) on memory retrieval was measured (a) at 4-h delay after acute ethanol administration, as well as (b) after acute withdrawal (24 and 48 h) of repeated (2.0 g/kg, once daily for 7 days) ethanol administration. Additionally, the effects of VU-29 on expression of mGlu5 and mGlu2 receptor proteins in the hippocampus, prefrontal cortex, and striatum were determined 48 h after ethanol withdrawal. Our results indicated that VU-29, given before acute ethanol administration, prevented the ethanol-induced impairments in spatial memory retrieval. Furthermore, VU-29 given before the testing session on the first day of abstinence facilitated NOR performance in ethanol-withdrawn rats at 4- and 24-h delay after administration. Our ELISA results show that VU-29 normalized ethanol withdrawal induced increase in expression of mGlu5 receptor protein in the hippocampus, prefrontal cortex, and striatum, as well as expression of mGlu2 receptor protein in the hippocampus. Thus, results from our study indicate that positive modulation of mGlu5 receptor prevented and reversed ethanol-induced memory impairment. Moreover, mGlu5 (hippocampus, prefrontal cortex, and striatum) and mGlu2 (hippocampus) receptors play an important role in the ethanol-induced recognition memory impairment induced by ethanol withdrawal.

## Introduction

Acute ethanol intoxication (Ryabinin 1998; White et al. 2000) and long-term ethanol consumption (Meyerhoff et al. 2004; Schweizer et al. 2006; Friedman et al. 2011) lead to a broad spectrum of cognitive deficits in humans. In animal studies, ethanol preferentially impairs a variety of hippocampal-dependent spatial memory tasks (Givens et al. 2000; Miki et al. 2000; White and Best 2000; Matthews and Morrow 2000; Chin et al. 2011; Gawel et al. 2016); although, it has also an impairing effect on spatial and non-spatial working memory (Santin et al. 2000; Ryabinin et al. 2002; Silvers et al. 2003; Berry and Matthews 2004) and on object recognition tasks which require non-spatial recognition memory (Brooks et al. 2002; Garcia-Moreno et al. 2002; Garcia-Moreno and Cimadevilla 2012; Yu et al. 2013; Zhao et al. 2013).

Recognition memory is the ability to recognize previously encountered events, objects, or people, and this memory depends on the integrity of the medial temporal lobe regions (Squire et al. 2007), which include the hippocampus and perirhinal cortex (Squire and Zola-Morgan 1991). As demonstrated before (Crews et al. 2004), these brain structures are very susceptible to ethanol-induced neurodegeneration. The perirhinal cortex is a crucial region for recognition memory performance (Brown et al. 1987; Zhu et al. 1995; Brown and Xiang 1998), but the role of the hippocampus in object recognition is still under debate (Mumby et al. 2002; Langston and Wood 2010; Clark et al. 2000; Broadbent et al. 2009; Cohen et al. 2013; Yi et al. 2016). Also, it has been indicated that some forms of recognition memory recruit the medial prefrontal cortex (Bekinschtein and Weisstaub 2014; Morici et al. 2015) that is associated with successful retrieval of the object recognition task (Bekinschtein and Weisstaub 2014; Morici et al. 2015).

It has been recently reported that metabotropic glutamate subtype 5 (mGlu5) receptor-dependent signaling, preferentially in limbic structures, is involved in cognition (e.g. memory and learning), novelty seeking behavior, and compulsivity. These factors are core personality traits increasing the susceptibility to drug addiction (Leurquin-Sterk et al. 2015). Indeed, it has been indicated the mGlu5 receptors are involved in nicotine addiction in humans (Akkus et al. 2012, 2016). Furthermore, preclinical researches suggest the important role of this receptor in ethanol drinking (Cozzoli et al. 2009, 2012; Besheer et al. 2010; Sinclair et al. 2012) and in the memory formation responsible for the chronic relapsing nature of alcohol abuse (Obara et al. 2009). Therefore, this receptor is a candidate target for the treatment of addictive disorders (Mihov and Hasler 2016).

In the present study, we examined the influence of the mGlu5 receptor-positive allosteric modulator, VU-29, on ethanol-induced impairment of recognition, in rats, using the novel object recognition (NOR) test. Thus, in experiment 1, we examined whether VU-29 given before the test was able to prevent the ethanol-induced impairment of the recognition memory. Thus, we first selected the ethanol dose that induced memory, but not motor impairment. Next, we used this dose in combination with VU-29 to indicate the influence of this compound on memory retrieval impaired by acute ethanol administration.

In a second experiment, we evaluated the influence of VU-29 on ethanol-induced memory retrieval impairment during the first 24- and 48-h of ethanol withdrawal. Group II mGlu (mGlu2/3) receptors in combination with the mGlu5 receptor and the *N*-methyl-d-aspartate (NMDA) receptor are important for recognition memory (Cho et al. 2000), and among the group II mGlu receptors, the mGlu2 receptor particularly plays an essential role in ethanol dependence and relapse (Meinhardt et al. 2013). Thus, we assessed the effect of ethanol withdrawal on potential changes in mGlu (mGlu5 and mGlu2) receptor protein expression after day 2 and day 7 of ethanol abstinence in brain areas important for memory retrieval such as the prefrontal cortex (Rugg et al. 2003; Bekinschtein and Weisstaub 2014; Morici et al. 2015), striatum (Scimeca and Badre 2012), and hippocampus (Clark et al. 2000; Broadbent et al. 2009; Cohen et al. 2013; Yi et al. 2016). Furthermore, we evaluated the influence of VU-29 pre-treatment on ethanol-induced changes in mGlu5 and mGlu2 receptor protein expressions in these brain structures after 2 days of withdrawal from repeated ethanol treatment. Also, we performed a study to exclude the effects of acute ethanol intoxication (locomotor effects) and ethanol withdrawal (anxiety) on the obtained results. The data we obtain could, therefore, have therapeutic implications for alcohol addiction.

## Materials and Methods

### Animals

Male naïve Wistar rats (HZL, Warsaw, Poland) weighing 200–250 g at the initiation of the experimental procedure (age of 8–9 weeks) were used in our experiments. A total of 136 animals (8–10/group) were used in our study. The rats were housed four per cage with a standard diet (Agropol, Motycz, Poland) and water ad libitum. They were kept under 12-h light/dark cycle and controlled temperature (22 ± 2 °C). The rats were adapted to the laboratory conditions for at least 1 week before the experiments and were handled twice daily for 5 days before the beginning of the tests. All behavioral studies were performed between 9:00 a.m. and 5:00 p.m. The experimental protocols and housing conditions were performed according to the National Institute of Health Guidelines for the Care and Use of Laboratory Animals, the European Community Council Directive of November 2010 for Care and Use of Laboratory Animals (Directive 2010/63/EU), and were approved by the Local Ethics Committee.

### Drugs

For acute injection, ethanol (95%, *w*/*v*) was diluted in saline (0.9% NaCl) to a concentration of 10% (*w*/*v*) and administered intraperitoneally (i.p.) 30 min prior to testing in three doses (1.5, 1.75, and 2.0 g/kg). This time of ethanol administration was selected (taking into account published data and our preliminary experiments) because peak blood ethanol expression following systemic injection of ethanol is obtained 20–45 min post injection, and doses of 1.5 and 2.0 g/kg ethanol corresponded to blood ethanol levels of ~ 125 mg% (Givens and Breese 1990) and of ~ 205 mg% (Devenport et al. 1989), respectively. For repeated administration, ethanol (95%, *w*/*v*) was diluted in saline to a concentration of 20% (*w*/*v*) and administered intragastrically (i.g.) (preferred route of chronic drug administration) at the dose of 2.0 g/kg (Gomez et al. 2013). Previous study has shown that this ethanol dose corresponds to blood ethanol levels of ~ 160 mg% (Gomez et al. 2013). Control rats received saline instead of ethanol. All rats were gavaged with ethanol or saline at the same time every day (8.00). For both routes of ethanol administration (i.p. and i.g.), injection volumes were 1 ml per 200 g body weight. The mGlu5 receptor-positive allosteric modulator *N*-(1,3-diphenyl-1H-pyrazolo-5-yl)-4-nitrobenzamide (VU-29) (Chen et al. 2008) (synthesized at Merz Pharmaceuticals GmbH, Frankfurt am Main, Germany) was dissolved in a vehicle consisting of 10% Tween-80 (Sigma) in saline and given at the dose of 30 mg/kg, i.p., in a value of 1 ml/kg, 90 min before test (I. Belozertseva, personal communication, as well as our preliminary study). This compound is a highly selective positive allosteric modulator of mGlu5 over mGlu1 and mGlu2 receptors (de Paulis et al. 2006) with promoting effect on hippocampal synaptic plasticity (Ayala et al. 2009).

### NOR Test

The NOR test was based on methods described previously (Reichel et al. 2011; Gomez et al. 2013) with modification of testing time (Davis et al. 2010). Shortly, the animals were handled twice daily for the week preceding testing. The test was carried out in the same Plexiglas square box (63 cm long × 44.5 cm high × 44 cm wide) in a dimly lit (30 ± 5 lx) testing room. All rats were given two 3-min sessions (a training session and a testing session). During the training session (the first session, T1A, familiarization phase), the rats explored two identical objects (A_1_ and A_2_). After a 4 h interval, during the testing session (T1B) (short-term memory), rats were allowed to explore one familiar object (A2) from the first session and a novel object (B) that was different enough in shape to be distinguished (Davis et al. 2010; Gomez et al. 2013). The objects were made of glass, plastic, and wood and were chosen after determining, in preliminary experiments with other animals, that they were equally preferred. Shapes, colors, and textures were different among these objects. Exploration of the objects was defined as sniffing or touching with the nose towards the object at a distance of less than 1 cm; however, sitting on the object was not considered exploration (Clark et al. 2000). After 24-h delay (long-term memory), the same rats underwent the second memory test without further drug treatment (Davis et al. 2010; Gomez et al. 2013; Reichel et al. 2011). In each test session, the rats were placed back in the box with one familiar and one novel object (C) and allowed to explore freely for a total of 3 min. Object placement was counterbalanced within each group in order to avoid any bias due to a preference that rats may have for a given object or its position in the box. The box and the objects were cleaned with 10% ethanol solution between each trial to remove traces of odor. A discrimination index (DI) was calculated, this being the ability to discriminate the novel from the familiar object: (novel object (s)/novel object (s) + familiar object (s) × 100%). Thus, an index > 50% indicates novel object preference, < 50%—familiar object preference, and 50%—no preference (Hammond et al. 2004).

### Locomotor Activity Test

To exclude locomotor activity influence on NOR results, the locomotor activity of individual rats was recorded using a photocell apparatus (Porfex, Bialystok, Poland). The animals were individually placed in Plexiglas boxes (square cages, 60 cm each side), in a sound-attenuated experimental room, under moderate illumination (5 lx). Ambulatory activity (distance traveled) was measured for 15 min by two rows of the infrared light-sensitive photocells located along the long axis, 45 and 100 mm above the floor. Locomotor activity test was conducted after acute ethanol (1.5, 1.75, 2.0 g/kg, i.p.) administration.

### Elevated Plus Maze Test

The elevated plus maze test (EPM test) was designed to test general anxiety levels in animal models (Pellow et al. 1985). The EPM apparatus was made of wood and positioned on a height of 50 cm above the floor. Two opposite arms were open (50 × 10 cm), and the other two were walled-off (50 × 10 × 25 cm). The experiment was carried out in a quiet room with constant light of 100 lx. Rats were tested on the EPM 24 h after the last ethanol administration (2.0 g/kg, 20% *w*/*v*, i.g., 1 for 7 days). The EPM experiment was performed by placing the rat in the center of the plus maze facing an open arm, after which the number of entries and time spent in the open arms were recorded for a period of 5 min. An “arm entry” was recorded when the rat entered the arm with all four paws into the arm. The open arms activity was quantified as follows: (A) the number of entries into the open arms as a percentage of the total number of entries into both open and closed arms and (B) the time spent in the open arms as a percent of the total time spent on exploring both the open and closed arms. Locomotor activity of rats (C) was measured as the total number of entries into the closed and open arms of the plus-maze apparatus. The EPM test was conducted 24 h after the last repeated ethanol administration—not only to test for anxiety, but also to test for any possible withdrawal effects of alcohol. Published data suggest that the NOR task conducted immediately after the EPM had no effect on rat performance (Gomez et al. 2013).

### Brain Dissection and Tissue Preparation

A separate group of rats was sacrificed by decapitation on day 2 or 7 of ethanol abstinence, and the brains were removed. The prefrontal cortex, hippocampus, and striatum were immediately dissected, and all samples were stored in a liquid nitrogen tissue bank (LS750, Taylor-Wharton, USA) until analyses. Next, the samples were mixed with 500 μl of cold phosphate buffered saline and homogenized on ice using a mechanical homogenizer (BioGen PRO200, PRO Scientific, Oxford, USA). Tip speed was set to ca. 15,000 rpm for 5 s. The samples were then frozen/defrozen twice to destroy remaining cell conglomerates and, subsequently, centrifuged (+ 4 °C, 15 min, 4500×*g*, centrifuge model 5804R, Eppendorf AG, Germany). To estimate protein content in supernatants, all samples were measured colorimetrically with the aid of the Bradford method.

The mGlu2 and mGlu5 receptor protein expressions were measured using ELISA kits: for mGlu2 receptor—kit number E02M0093 (receptor’s standard concentration range from 0.5 to 10 ng/ml) and for mGlu5 receptor—kit number E02M0097 (receptor’s standard concentration range from 0.5 to 10 ng/ml), both from BlueGene Biotech Corp., Shanghai, China. Measurement was done using an ELISA spectrophotometric reader: SpectrostarNano (BMG Labtech, UK), according to the manufacturer’s protocols.

### Experimental Procedure

- Experiment 1. The influence of acute ethanol administration on NOR memory after 4-h delay and locomotor activity in rats. The effect of VU-29 on ethanol-induced memory impairment

**Fig. 1:**
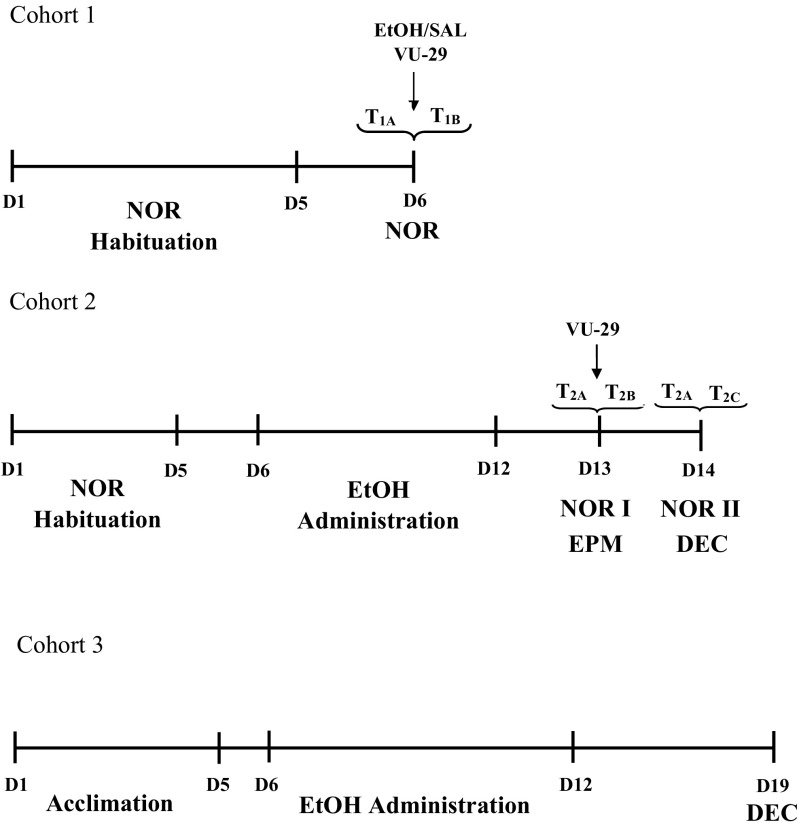
Timeline for experiment 1 (cohort 1) and experiment 2 (cohorts 2 and 3). Each tick mark represents 1 day. Longer tick marks represent the beginning or ending of the experiment phase. D day, EtOH ethanol, SAL saline, LOC locomotor activity, EPM elevated plus-maze, NOR novel object recognition, DEC decapitation

After acclimation to the environment, rats (cohort 1) were habituated to the NOR apparatus for 5 days (Gomez et al. 2013). On day 1, rats were exposed to the testing apparatus for 5 min/session (no object). From days 2–5, once a day for 5 min per session/day, the animals were habituated to the testing apparatus which contained two selected objects (these objects were not used in the following test trials). Next, 24 h after the last session of habituation, the test session for examining object memory was given. This test was based on two 3-min sessions: a training session (T1A) and a testing session (T1B), separated by 4-h delay. Before the testing session, the rats received either acute ethanol administration (10% *w*/*v*) at the dose of 1.5, 1.75, and 2.0 g/kg, i.p. or saline, 30 min prior the trial. Immediately after the NOR test, the influence of ethanol (1.5–2.0 g/kg, i.p.) on locomotor activity (locomotor activity cages) was assessed. This was done to exclude the influence of the ethanol doses on locomotor disturbances so as to exclude nonspecific effects in the memory task. On the basis of this experiment, the dose of ethanol of 1.75 g/kg was selected to determine the effect of mGlu5 receptor-positive allosteric modulator, VU-29, on memory retrieval impaired by acute ethanol administration. Thus, the rats received VU-29 (30 mg/kg, i.p.), 90 min prior to the testing session (60 min prior to ethanol at the dose of 1.75 g/kg), and a T1B session was conducted according to the procedure described above (see Fig. 1a). An observer naive to the experimental conditions manually scored behavior.

- Experiment 2. The influence of VU-29 on NOR memory impaired by repeated ethanol administration in NOR test after 4- and 24-h delay: mGlu5 receptor and mGlu2 receptor expressions in the cortex, hippocampus, and striatum

After 5 days of habituation to the NOR apparatus, the rats (cohort 2) received ethanol (20% *w*/*v*) once daily at the dose of 2.0 g/kg, i.g., for 7 days (Gomez et al. 2013). Next, during the training session (T2A), the rats explored two identical objects for 3 min (day 1 abstinence), and the first memory testing session (T2B) was conducted after a 4-h delay, by allowing the rats to explore an object from the training session, as well as a novel object, for 3 min. After 24-h delay (day 2 abstinence), the second memory testing session (T2C) was conducted (performed 4-h follow the first training session). In this, the rats were given the familiar object and a different novel object. VU-29 was injected at the dose of 30 mg/kg, i.p., 90 min prior to the first testing session (T2B), on the first day of ethanol abstinence. Subsequently, the groups of rats (*N* = 8–9) were rapidly decapitated and their brains were removed on the second day of abstinence (after T2C session) (see Fig. 1b). Furthermore, two additional groups (*N* = 8) of rats (cohort 3) that received ethanol (20% *w*/*v*) once daily at the dose of 2.0 g/kg, i.g., or saline for 7 days were decapitated on the seventh day of abstinence (see Fig. 1c). After this, the expressions of mGlu5 receptor and mGlu2 receptor in the cortex, hippocampus, and striatum were established.

### Statistical Analysis

**Fig. 2:**
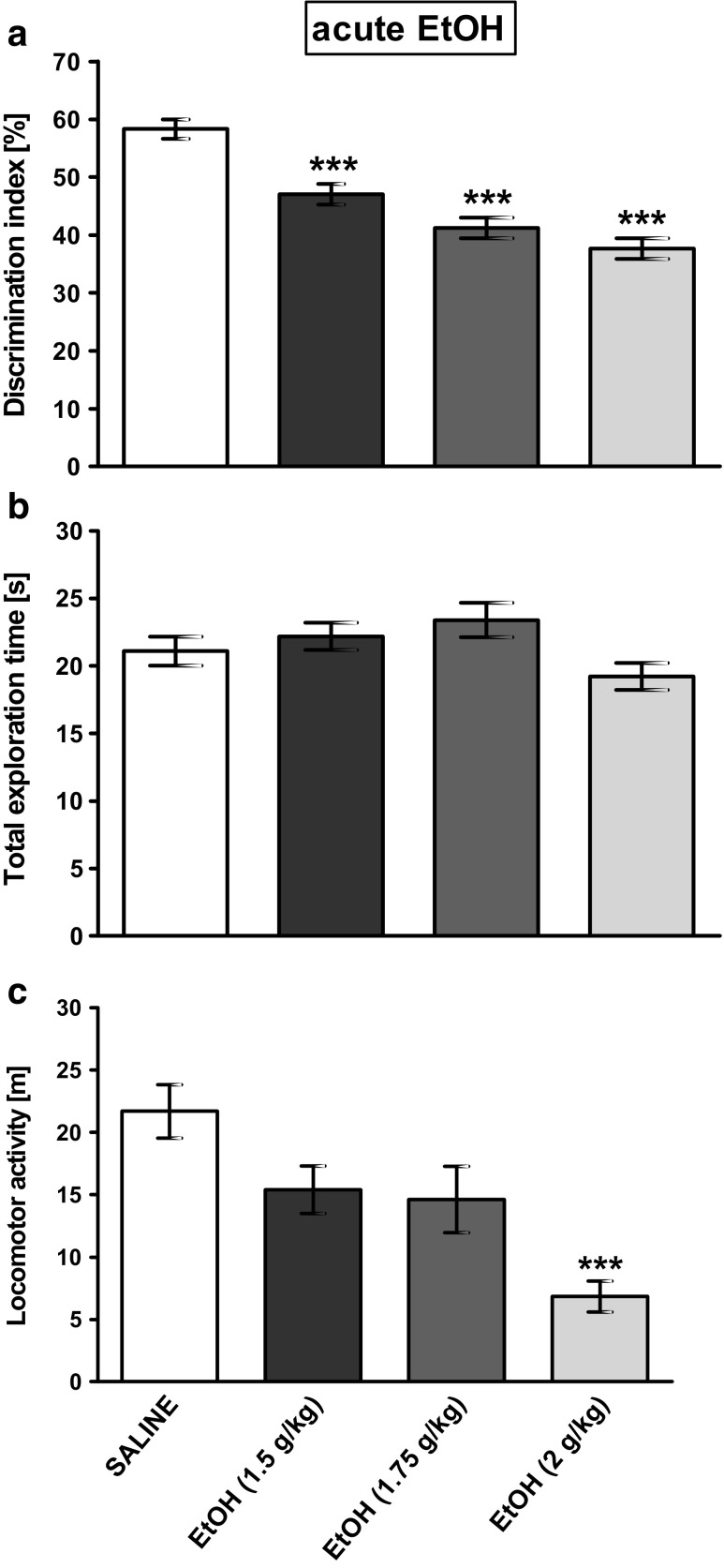
The influence of acute ethanol administration on **a** memory performance (expressed as a discrimination index) in the NOR test in rats. **b** Total exploration time displayed by different groups of rats in the NOR test, in T1B. **c** Locomotor activity. Ethanol was administered at the dose of 1.5, 1.75, and 2.0 g/kg (10%, *w*/*v*, i.p.) 30 min before T2B. Each column represents the mean ± SEM (*N* = 9–10). ***P < 0.001 vs. vehicle group

**Fig. 3:**
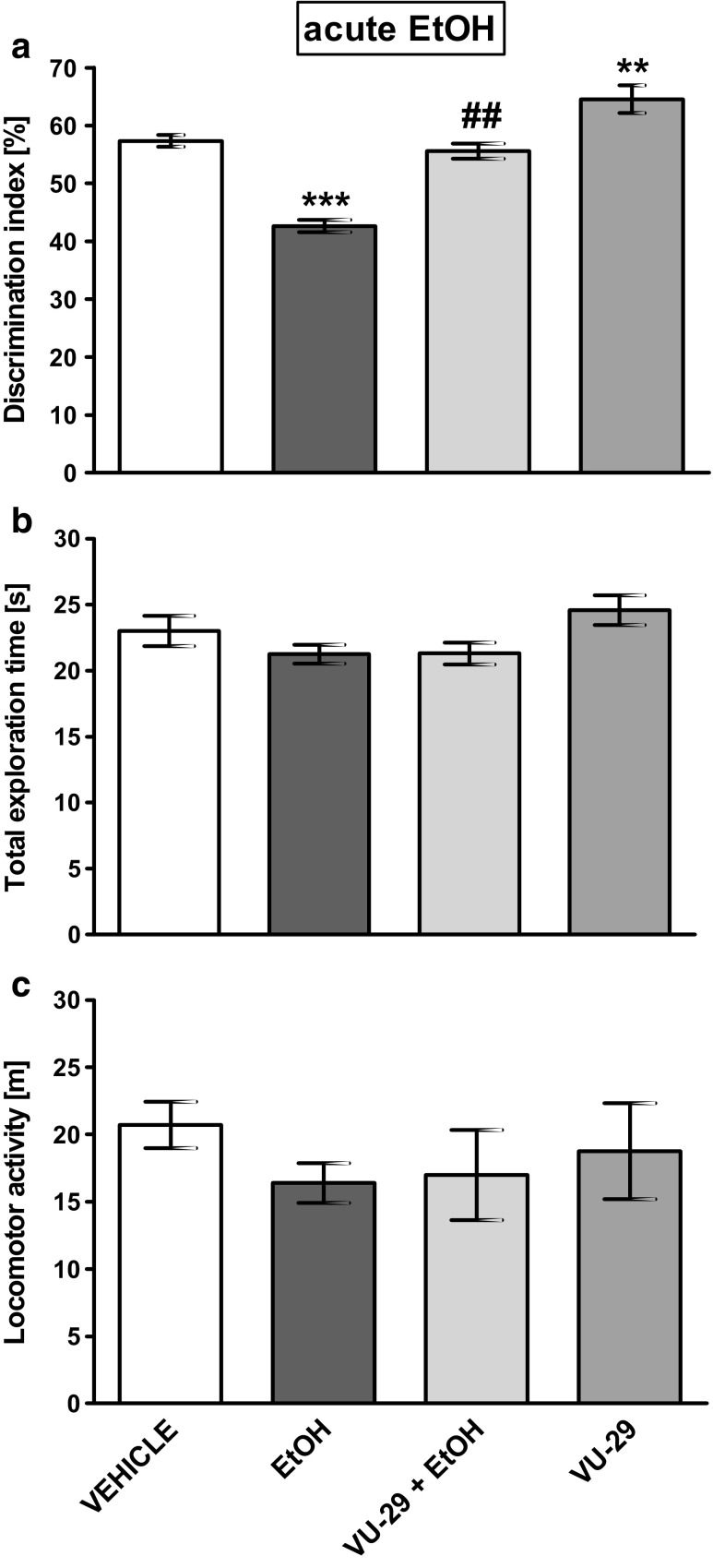
The influence of VU-29 on (**a**) memory performance (expressed as a discrimination index) induced by acute ethanol administration in the NOR test in rats. (**b**) Total exploration time displayed by different groups of rats in the NOR test, in T2B. (**c**) Locomotor activity. Ethanol was administered at the dose of 1.75 g/kg (10%, *w*/*v*, i.p.) 30 min before T2B and VU-29 was injected at the dose of 30 mg/kg, i.p., 90 min before T2B. Each column represents the mean ± SEM (*N* = 9–10). **P < 0.01, ***P < 0.001 vs. vehicle group, ^*##*^*P* < 0.01 vs. ethanol-treated group

**Fig. 4:**
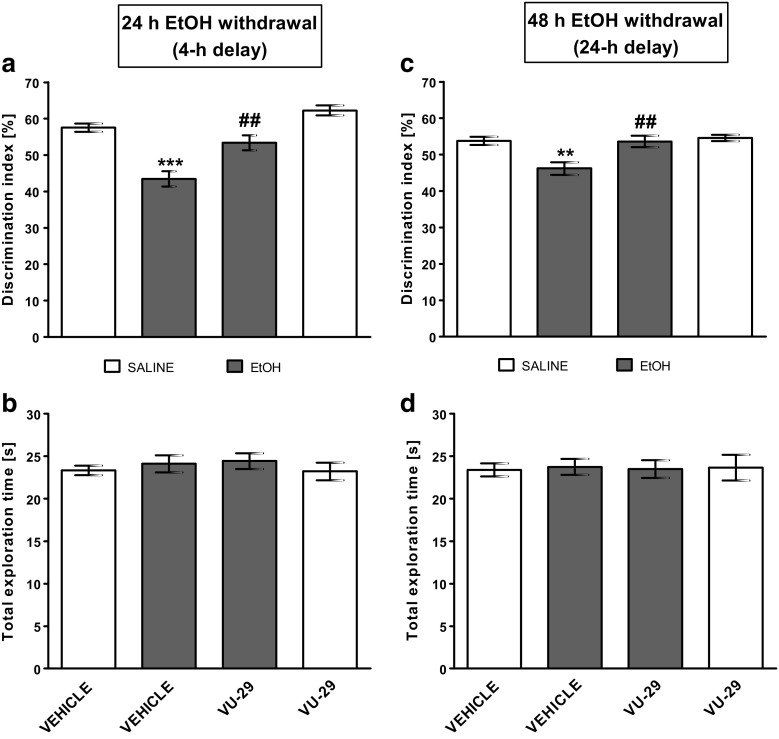
The influence of VU-29 on memory performance (expressed as a discrimination index) induced by repeated ethanol administration at (**a**) 24 and (**c**) 48 h post-treatment in the NOR test in rats. Total exploration time displayed by different groups of rats in the NOR test (**b**, **d**). Ethanol was administered at the dose of 2.0 g/kg (20% *w*/*v*, i.g.) for 7 days. VU-29 was injected at the dose of 30 mg/kg, i.p., 90 min before T2B on the first day (24 h) of ethanol withdrawal. Each column represents the mean ± SEM (*N* = 8–9). **P < 0.01, ***P < 0.001 vs. vehicle group, ^*##*^*P* < 0.01 vs. ethanol-withdrawal group

The obtained results were analyzed using GraphPad Prism version 5.00 for Windows, GraphPad Software, San Diego, California, USA. Statistical significance of the data was assessed by a one-way (one factor with four levels, corresponding to four groups shown in Fig. 2: saline and three (1.5, 1.75, and 2 g/kg) concentrations of ethanol) and two-way (two factors in four possible combinations as shown in Figs. 3 and 4: ethanol presence and VU-29 presence) analysis of variance (ANOVA). *Post-hoc* comparisons were carried out with the Tukey’s (for one-way ANOVA) or with the Bonferroni’s (for two-way ANOVA) test. The Student’s *t* test was used to analyze EPM data. The value of *P* < 0.05 was considered statistically significant. Data were reported as mean ± standard error of the mean (SEM) of 8–10 subjects per group.

## Results

In the NOR test during the training session (T1), the subject rats spent a similar period of time exploring two identical objects. Statistical analysis showed no significant difference in the exploration of the identical objects (A_1_ and A_2_) during this phase, in any of the treatment groups (data not shown). However, the DI was significantly larger than 50% in saline groups: (Fig. 2: *t* = 4.954, df = 9, *P* = 0.0003934856; Fig. 3: *t* = 7.325, df = 9, *P* = 2.222113e−05; Fig. 4a: *t* = 3.094, df = 9, *P* = 0.006423126; Fig. 4c: *t* = 2.185 df = 9, *P* = 0.02835527). This means that all saline groups successfully learned to discriminate the novel object.

### The Influence of Acute Ethanol Administration on NOR Memory After a 4-h Delay, in Rats

Figure 2 shows the effect of acute ethanol (1.5, 1.75, and 2.0 g/kg) administration before T1B on DI after a 4-h retention delay. One-way ANOVA revealed a significant effect (*F*(3,35) = 26.95, *P* < 0.001; *N* = 9–10/group) (Fig. 2a). Post-hoc test showed that acute ethanol administration at all used doses, 30 min before the testing session (T1B) impaired novel object recognition (*P* < 0.001), as revealed by significantly lower DI in ethanol groups relative to the saline group (Fig. 2a). Analysis of the total exploration times during T1B session did not show a statistically significant differences between groups (*F*(3,35) = 2.512, *P* = 0.074) (Fig. 2b). However, one-way ANOVA revealed significant differences in the locomotor activity (*F*(3,35) = 9.349, *P* < 0.001) between group of rats. Post-hoc test indicated that acute ethanol administration significantly decreased at the dose of 2.0 g/kg (*P* < 0.001), but at the doses of 1.75 and 1.5 g/kg (30 min before the test), it indicated only trend towards decreased locomotor activity compared to that of the saline-treated group (Fig. 2c); therefore, the dose of 1.75 g/kg of ethanol was chosen for future study.

### The Influence of VU-29 on the NOR Memory Deficits in Rats Induced by Acute Ethanol Administration, After a 4-h Delay

Figure 3 shows the effect of VU-29 on DI impaired by acute ethanol (1.75 g/kg) administration, after a 4-h retention delay. Two-way ANOVA revealed the first factor (VU-29 vs. its absence) as statistically significant: (*F*(1,34) = 40.38, *P* < 0.0001; *N* = 9–10/group) and the second factor (ethanol vs. its absence) (*F*(1,34) = 55.56, *P* < 0.0001), also with significant interaction between these factors (*F*(1,34) = 3.29, *P* = 0.078). Post-hoc test showed that acute ethanol administration (1.75 g/kg) 30 min before the testing session (T1B) decreased DI, when compared to the saline-treated group (*P* < 0.001). Notably, VU-29 (30 mg/kg, i.p.) pre-treatment 60 min before ethanol decreased this deficit, as the VU-29/ethanol group showed a significantly increased DI (*P* < 0.01) when compared to ethanol group, as did the VU-29/saline group (*P* < 0.01) (Fig. 3a). However, DI in the VU-29/ethanol group was significantly (*P* < 0.0001) lower than those of the control (saline) group. Analysis of the total exploration times during T1B did not show a statistically significant interaction between pre-treatment (*F*(1,34) = 0.68, *P* = 0.416), treatment (*F*(1,34) = 6.34, *P* = 0.017), or pre-treatment × treatment interaction (*F*(1,34) = 0.60, *P* = 0.445) (Fig. 3b). In addition, no differences in locomotor activity was observed between treated animals (pre-treatment: *F*(1,34) = 0.07, *P* = 0.792; treatment: *F*(1,34) = 1.37, *P* = 0.250; pretreatment × treatment interaction: *F*(1,34) = 0.24, *P* = 0.628) (Fig. 3c).

### The Influence of VU-29 on the NOR Memory Deficits During Ethanol Abstinence in Rats, After a 4- and a 24-h Delay

After 24 h of ethanol abstinence, during the testing session (T2B) (after a 4-h retention delay), two-way ANOVA indicated statistically significant differences between pre-treatment (ethanol effect) (*F*(1,31) = 25.25, *P* < 0.0001; *N* = 8–9/group), treatment (VU-29 effect) (*F*(1,31) = 8.38, *P* = 0.007), and pre-treatment × treatment interaction (*F*(1,31) = 4.75, *P* = 0.037). Post-hoc test showed that the ethanol withdrawal rats discriminated worse the novel object than did the saline-treated group (*P* < 0.001). Moreover, a single injection of VU-29 (30 mg/kg, i.p.) 90 min before the testing session (T2B) significantly increased the DI in ethanol-treated animals (*P* < 0.01) (Fig. 4a) but only exhibited tendency to increase the DI (*P* > 0.05) in saline-treated rats. Furthermore, the DI in the VU-29/ethanol group was not significantly different from the saline group (*P* = 0.0819). Also, analysis of the total exploration times during T2B did not show a statistically significant interaction between pre-treatment (*F*(1,31) = 1.25, *P* = 0.271), treatment (*F*(1,31) = 0.01, *P* = 0.908), or pre-treatment × treatment interaction (*F*(1,31) = 0.06, *P* = 0.812) (Fig. 4b).

After 48 h of ethanol abstinence, during the testing session (T2C) (after a 24-h retention delay), two-way ANOVA indicated statistically significant differences between pre-treatment (ethanol effect) (*F*(1,31) = 10.83, *P* = 0.003; *N* = 8–9/group), treatment (VU-29 effect) (*F*(1,31) = 10.01, *P* = 0.004), and pre-treatment × treatment interaction (*F*(1,31) = 6.54, *P* = 0.016). Post-hoc test showed that animals during withdrawal from repeated ethanol discriminated worse the novel object than did the saline-treated group (*P* < 0.01). Furthermore, VU-29, given on the first day of ethanol abstinence (90 min before T2B), prevented (*P* < 0.01) this ethanol withdrawal-induced deficit in recognition memory (the DI was not significantly different from the saline group, *P* = 0.9298) as observed 48 h after cessation of ethanol treatment (24-h delay) (Fig. 4c). In this set of experiments, analysis of the total exploration times during T2C did not show a statistically significant interaction between pre-treatment (*F*(1,31) = 0.01, *P* = 0.934), treatment (*F*(1,31) = 0.00, *P* = 0.994), or pre-treatment × treatment interaction (*F*(1,31) = 0.05, *P* = 0.817) (Fig. 4d).

### Influence of Repeated Ethanol Administration on Anxiety-Like Behavior

Student’s *t* test indicated that there were no significant differences between repeated ethanol (2.0 g/kg, for 7 days) and saline administration on the number of open arm entries (*t* = 0.875, *P* = 0.396; *N* = 8) and the percent of time spent by rats in the open arm compartment of the maze (*t* = 0.487, *P* = 0.633) (Table 1). Ethanol withdrawal also did not produce any changes in locomotion of rats measured as the total number of entries into both the open and closed arms of the apparatus (*t* = 0.242, *P* = 0.812). Thus, treatment with ethanol had no effect on anxiety-like behaviors as compared to the control group (Table 1).

**Table 1:** The effect of repeated ethanol administration (2.0 g/kg, i.g., 1 for 7 days) on anxiety-like behavior in the EPM test on the first day of ethanol withdrawal: (A) percent of entries into the open arms; (B) percent of time spent in the open arms; (C) locomotor activity measured as a total number of closed and open arms entries. Each column represents the mean ± SEM (*N* = 8)

	Vehicle	EtOH
% Time in open arms	35.33 ± 9.255	40.75 ± 6.123
% Entries in open arms	36.74 ± 6.006	42.85 ± 3.575
Number of total entries	9.75 ± 0.796	9.50 ± 0.654

### Influence of Repeated Ethanol Administration on mGlu5 Receptor Protein Expression in the Cortex, Hippocampus, and Striatum After 2 and 7 days of Withdrawal. Effect of VU-29 on mGlu5 Protein Expression at Day 2 of Ethanol Withdrawal

**Fig. 5:**
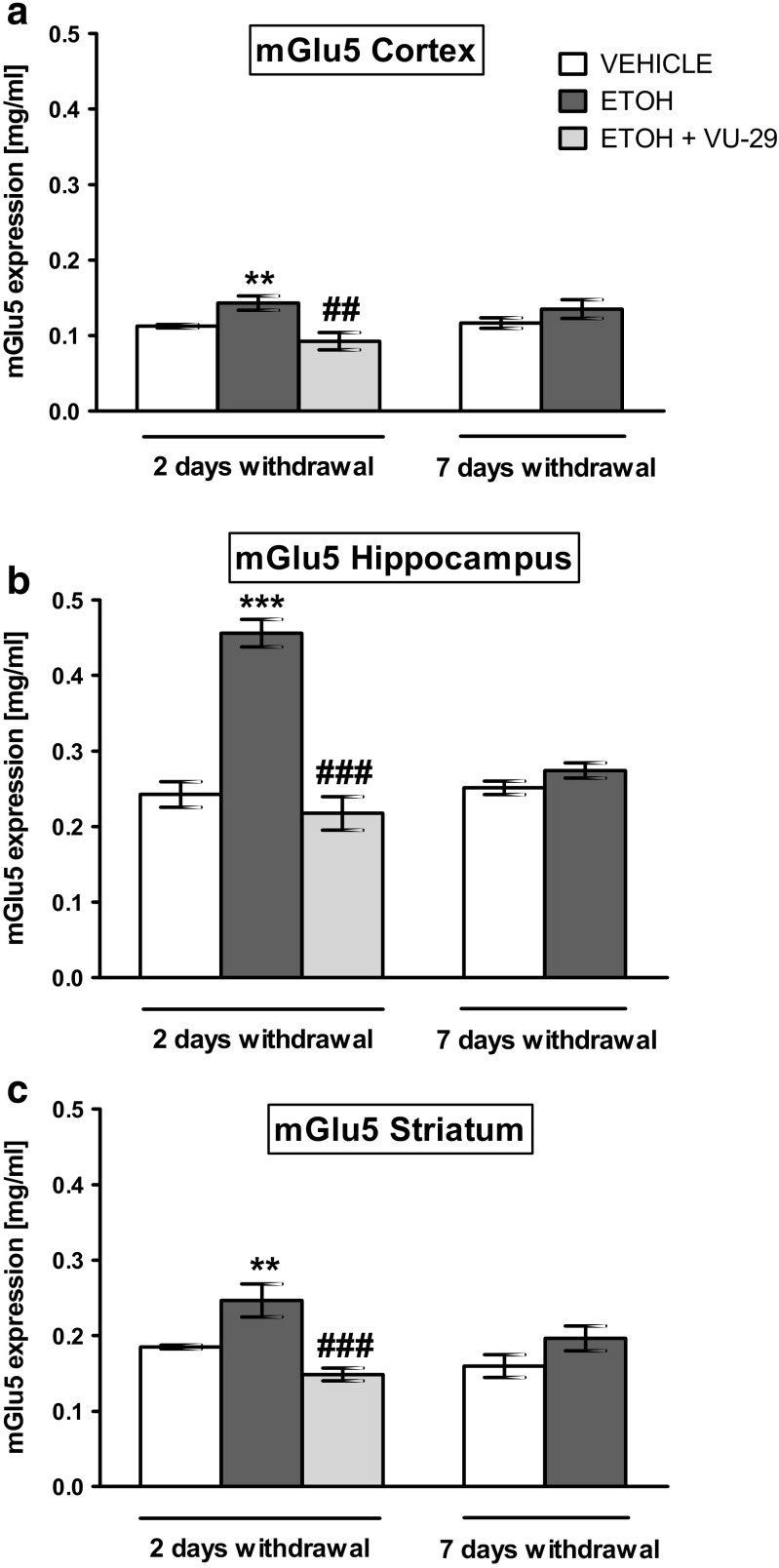
The effect of ethanol and VU-29 on mGlu5 levels in (**a**) the cortex, (**b**) hippocampus, and (**c**) striatum 2 and 7 days after last ethanol injection. Ethanol was administered at the dose of 2.0 g/kg (20% *w*/*v*, i.g.) for 7 days. VU-29 was injected at the dose of 30 mg/kg, i.p., 90 min before T2B on the first day (24 h) of ethanol withdrawal. Each column represents the mean ± SEM (*N* = 8–9). **P < 0.01, ***P < 0.001 vs. vehicle group, ^*##*^*P* < 0.01, ^*###*^*P* < 0.001 vs. ethanol-withdrawal group

Two-way ANOVA indicated statistically insignificant differences in the cortex between treatment factor (saline and ethanol) (*F*(1,32) = 0.05; *P* > 0.05), time factor (2 and 7 days of ethanol withdrawal) (*F*(1,32) = 8.64; *P* = 0.0064), and without significant interactions between these two factors (*F*(1,32) = 0.52; *P* > 0.05). Post-hoc test showed that repeated ethanol administration (2.0 g/kg, 20% *w*/*v* × 7 days) induced a statistically significant increase in mGlu5 receptor protein expression in the cortex after 2 days of ethanol withdrawal (*P* < 0.01), compared to the saline-treated rats (Fig. 5a). Furthermore, two-way ANOVA indicated statistically significant differences in the hippocampus between treatment (saline and ethanol) (*F*(1,32) = 80.94; *P* < 0.0001), time (2 and 7 days of ethanol withdrawal) (*F*(1,32) = 29.37; *P* < 0.0001), and exposing also significant interactions (*F*(1,32 = 38.63; *P* < 0.0001). Repeated ethanol administration induced a statistically significant increase in mGlu5 receptor protein expression in the hippocampus at 2 days of ethanol withdrawal (*P* < 0.001), when compared to the saline-treated rats (Fig. 5b). Moreover, two-way ANOVA indicated statistically significant differences in the striatum time (2 and 7 days of ethanol withdrawal) (*F*(1,32) = 20.55; *P* < 0.0001). Repeated ethanol administration induced statistically significant increases in mGlu5 protein expression in the striatum after 2 days of ethanol withdrawal (*P* < 0.001), when compared to the saline-treated rats (Fig. 5c).

Furthermore, one-way ANOVA indicated statistically significant effects of single VU-29 injection on mGlu5 receptor expression in the cortex (*F*(2,25) = 7.55; *P* = 0.0027) (Fig. 5a), hippocampus (*F*(2,25) = 49.08; *P* < 0.0001) (Fig. 5b), and striatum (*F*(2,25) = 23.97; *P* < 0.0001) (Fig. 5c) after 2 days of ethanol withdrawal. The post-hoc test showed that VU-29 (30 mg/kg, i.p.) given 90 min prior the testing trial on the first day of ethanol abstinence, decreased mGlu5 receptor protein expression in the cortex (*P* < 0.01) (Fig. 5a). Administration of VU-29 to the ethanol group (VU-29/ethanol group) significantly decreased mGlu5 protein expression vs. ethanol group (*P* = 0.0045). Herein, the protein expression was not significantly different from the saline group (*P* = 0.1360). The post-hoc test showed that VU-29 (30 mg/kg, i.p.), given 90 min prior the testing trial on the first day of ethanol abstinence, decreased mGlu5 receptor protein expression in the hippocampus (*P* < 0.001) (Fig. 5b). Protein expression in the VU-29/ethanol group did not differ significantly from the control (saline) group (*P* = 0.3790). The post-hoc test showed that VU-29 (30 mg/kg, i.p.), given 90 min prior the testing trial on the first day of ethanol abstinence, decreased mGlu5 receptor protein expression in the striatum (*P* < 0.001) (Fig. 5c) on the second day, when compared to ethanol-withdrawal rats. The mGlu5 protein expression in the VU-29/ethanol group differed significantly from the control (saline) group (*P* = 0.0009).

### The Effect of Ethanol and VU-29 on mGlu2 Receptor Protein Expression in the Cortex, Hippocampus, and Striatum at 2 and 7 days of Ethanol Withdrawal

**Fig. 6:**
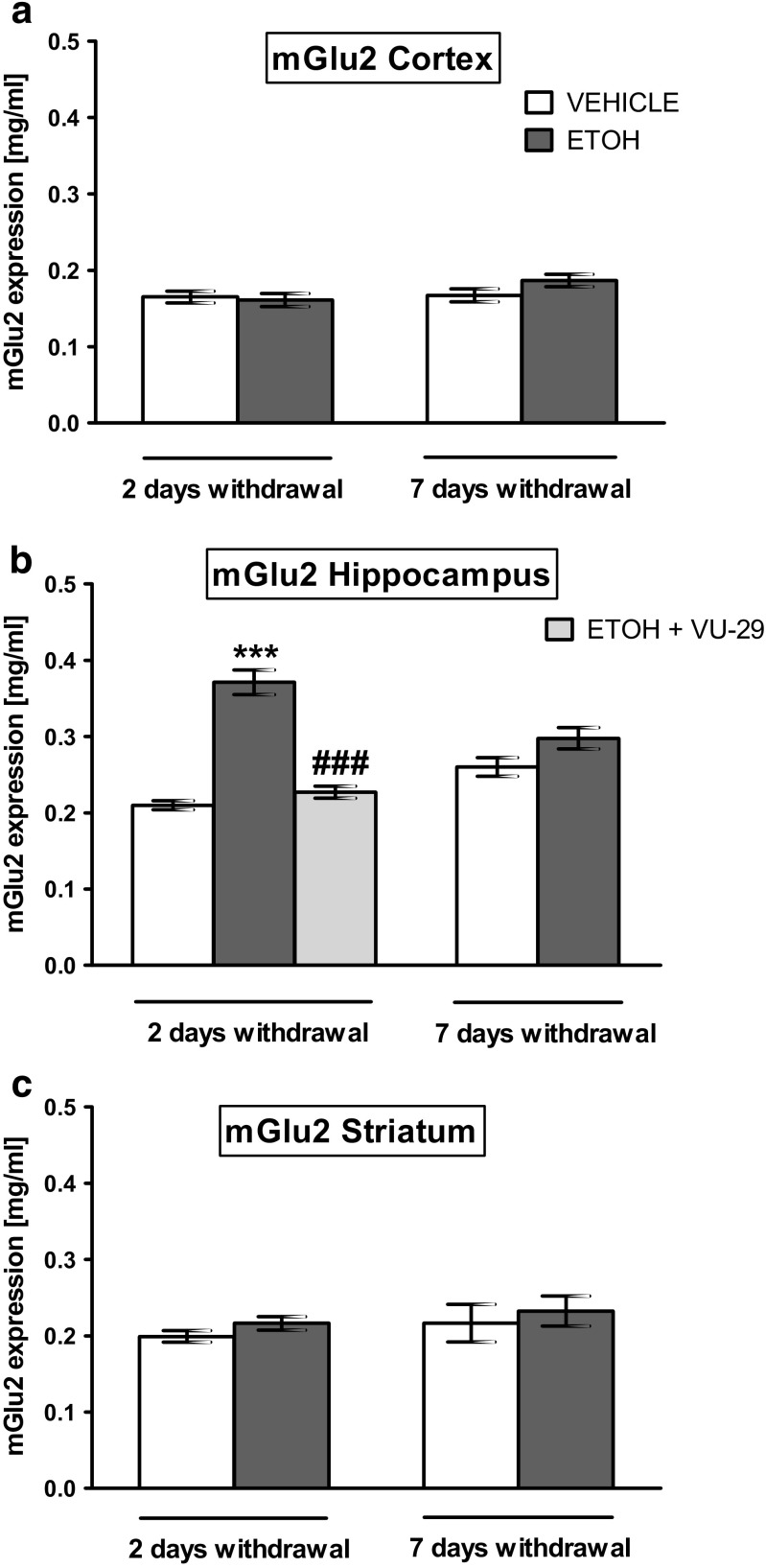
The effect of ethanol (**a**, **b**, **c**) and VU-29 (**b**) on mGlu2 levels in the cortex, hippocampus and striatum 2 and 7 days after last ethanol injection. Ethanol was administered at the dose of 2.0 g/kg (20% *w*/*v*, i.g.) for 7 days. VU-29 was injected at the dose of 30 mg/kg, i.p., 90 min before T2B on the first day (24 h) of ethanol withdrawal. Each column represents the mean ± SEM (*N* = 8–9). ***P < 0.001 vs. vehicle group, ^*###*^*P* < 0.001 vs. ethanol-withdrawal group

Two-way ANOVA did not indicate statistically significant differences in mGlu2 receptor protein expression in the cortex between treatment (saline and ethanol) (*F*(1,32) = 4.01; *P* > 0.05), time (2 and 7 days of ethanol withdrawal) (*F*(1,32) = 0.11; *P* > 0.05), and treatment × time interaction (*F*(1,32) = 0.05; *P* > 0.05) (Fig. 6a). However, two-way ANOVA did indicate statistically significant differences in the hippocampus between treatment (saline and ethanol) (*F*(1,32) = 0.71; *P* > 0.05), time (2 and 7 days of ethanol withdrawal) (*F*(1,32) = 62.80; *P* < 0.0001), and treatment × time interaction (*F*(1,32) = 21.35; *P* < 0.0001) (Fig. 6b). Moreover, post-hoc test showed that repeated ethanol administration (2.0 g/kg, 20% *w*/*v* × 7 days) brought a statistically significant increase in mGluR2 receptor protein expression in the hippocampus 2 days of ethanol withdrawal (*P* < 0.001), compared to saline-treated rats (Fig. 6b). Still, two-way ANOVA did not indicate statistically significant differences in the striatum between treatment (saline and ethanol) (*F*(1,32) = 3.22; *P* > 0.05), time (2 and 7 days of ethanol withdrawal) (*F*(1,32) = 0.80; *P* > 0.05), and treatment × time interaction (*F*(1,32) = 0.69; *P* > 0.05) (Fig. 6c).

One-way ANOVA indicated statistically significant differences between mGlu2 receptor protein expression in the hippocampus, in the ethanol withdrawal, and in control (saline) groups, on the second day (day 2) after single injection of VU-29 (*F*(2,24) = 50.34; *P* < 0.0001) (Fig. 6b). What is more, post-hoc test showed that acute injection of VU-29 (30 mg/kg, i.p.) on the first day of ethanol withdrawal, 90 min prior to the testing trial, decreased mGluR2 receptor protein expression in the hippocampus 24 h later (*P* < 0.001) (Fig. 6b) but the results did not differ significantly from the saline group (*P* = 0.0980).

## Discussion

Our study shows that acute ethanol administration, prior to the testing session, impaired retrieval of the recognition memory in the NOR task in rats. Animals treated with acute ethanol failed to recognized a novel object during the test phase. Moreover, pretreatment with VU-29, a positive allosteric modulator of mGlu5 receptor, before ethanol administration, significantly decreased ethanol-induced memory impairment. Furthermore, repeated (1× for 7 days) ethanol administration also caused NOR memory deficits in the acute (24–48 h) withdrawal phase. These cognitive deficits were accompanied by alterations in hippocampal expression of mGlu5 and mGlu2 receptors proteins, and to a lesser extent, in expression of cortical and striatal mGlu5 receptor protein during acute ethanol withdrawal (day 2 of abstinence). The casual relationship between these events remains to be demonstrated. Single administration of VU-29 prior to the testing session on the first abstinence day recovered the impaired recognition memory measured at both 4-h delay (short-term memory) and at 24 h-delay (long-term memory) intervals (the second day of abstinence). Our ELISA assay show that such VU-29 administration returned to control values the elevated mGlu5 receptor protein expressions in the hippocampus and prefrontal cortex, as well as mGlu2 receptor protein expressions in the hippocampus at 48 h of ethanol abstinence.

### Effect of VU-29, a mGlu5 Receptor-Positive Allosteric Modulator, on the Acute Effect of Ethanol on Retrieval of Recognition Memory

Preclinical studies suggest that acute ethanol administration has deleterious effects on recognition memory (Brooks et al. 2002; Ryabinin et al. 2002; Garcia-Moreno and Cimadevilla 2012; Yu et al. 2013), especially when it is administered before the training session of the test. Our experiments in rats extended these findings and indicated that acute ethanol administration induced a deficit in memory retrieval in the NOR test. Analysis of animal behavior showed that rats injected with ethanol at the doses tested (1.5, 1.75, and 2 g/kg) before the testing session demonstrated decreased preference for a novel object. Thus, ethanol produced retrograde amnesia for previously acquired information, including information about familiarity of objects previously encountered. Our experiments also indicated that ethanol at the dose of 2 g/kg significantly decreased locomotor activity, and, at lower doses (1.5 and 1.75 g/kg), showed a trend towards decreasing it. However, the total exploration time of the objects (sniffing or touching with the nose towards the object) for the rats did not differ significantly between the groups. Thus, our data support other research that reveals that there is clear dissociation between locomotor activity and exploration, and no correlation exist between these two measures. Such results underline the distinction for the two behaviors (Leyland 1976). Because exploration time generates information about the object via direct (animal) contact with it and this type of movement is important for the novel object recognition, but locomotor activity concerns movement from one location to other and could also be important for our study, we choose such dose of ethanol (1.75 g/kg) for our study that did not change significantly the locomotor activity.

Published data indicated that mGlu5 receptors are found in brain regions important for cognition, including the prefrontal cortex, striatum, and hippocampus (Packard et al. 2001; Melendez et al. 2004; Manahan-Vaughan and Braunewell 2005). Systemic administration of mGlu2/3 receptor, as well as mGlu5 receptor antagonists, before novel objects training and before testing, impaired long- (Barker et al. 2006a, b) and short-term recognition memories or retrieval of such memories (Christoffersen et al. 2008). In turn, stimulation of mGlu5 receptor via positive allosteric modulators (CDPPB or ADX47273) enhanced recognition memory in normal rats and mice (Liu et al. 2008; Uslaner et al. 2009; Fowler et al. 2013). Published data indicated that VU-29 (Ayala et al. 2009) potentiated long-term potentiation (LTP) or long-term depression (LTD) in hippocampal slices in vitro, both widely accepted processes of memory formation (Neyman and Manahan-Vaughan 2008). In our study, VU-29, a mGlu5 receptor-positive allosteric modulator, given before the test enhanced recognition memory in control rats. Furthermore, a single pretreatment with VU-29 decreased NOR deficit induced by acute ethanol administration. It should be noted that mGlu5 receptor is physically linked to NMDA receptors via several anchoring proteins, including Homer proteins (Fagni et al. 2004), and mGlu5 receptor activation can overcome or prevent the consequences of inhibition of NMDA receptors. As previously stated, acute ethanol administration inhibits, or antagonizes, the action of the NMDA receptors in several brain regions (Dildy and Leslie 1989; Hoffman et al. 1989; Lovinger et al. 1989; Simson et al. 1991). Thus, in our study, the protective effect of VU-29, a mGlu5 receptor-positive allosteric modulator on ethanol-induced memory deficit, might occur via the ability of this agent to decrease the consequences of ethanol-induced inhibition of the NMDA receptor.

### Effect of VU-29, a mGlu5 Receptor-Positive Allosteric Modulator, on Retrieval of Recognition Memory Impaired by Withdrawal from Repeated Ethanol Administration

After the first 24–48 h of withdrawal from repeated ethanol treatment (2.0 g/kg, i.g. for 7 days, once daily), the rats showed significant NOR memory deficits. Unlike the controls, rats treated with ethanol failed to recognize a novel object during the testing sessions. With the procedure used in this experiment, it is difficult to determine if the decreased novel subject exploration on the test day was due to a deficit in initial learning or in retrieval during the test. This effect was observed with no symptoms of withdrawal such as anxiety. However, published data (using other procedures of object recognition tests) have shown that withdrawal from other abuse drugs such as cocaine or methamphetamine also produce deficits in recognition (Briand et al. 2008; Rogers et al. 2008; Reichel et al. 2011) and CDPPB, a mGlu5 receptor-positive allosteric modulator, and reversed deficits in recognition memory during methamphetamine abstinence in rats (Reichel et al. 2011). In our study, VU-29, given 90 min before the testing session on the first day of ethanol abstinence, facilitated retrieval of NOR performance after a 4-h delay. This effect was long-lasting since it was also observed after a 24-h delay (Fig. 4).

Herein, ethanol withdrawal-induced deficits in the NOR test were associated with increased mGlu5 receptor protein expression at 48 h of abstinence, in brain structures important for retrieval of recognition memory, such as the hippocampus, prefrontal cortex, and striatum. VU-29 administration reversed this ethanol effect in the hippocampus and prefrontal cortex, but only decreased it in the striatum. Thus, we hypothesize that the positive allosteric modulator, VU-29 (given on the first day of ethanol withdrawal), potentiated mGlu5 receptor function by acting at allosteric sites and shifting the concentration response for glutamate to the left, without the risk of inducing excitotoxicity (Gass and Olive 2009). Still, a mGlu5 receptor-positive allosteric modulator can stabilize a conformation of the receptor (Homayoun and Moghaddam 2010) that would facilitate activation of neuroprotective pathways against neurotoxicity induced by glutamate release during withdrawal. A preventive effect of mGlu5 receptor-positive allosteric modulators against glutamate overstimulation of NMDA receptors was earlier described (Ribeiro et al. 2010; Doria et al. 2013). We put forward that such stabilization of neuronal function by VU-29 could have normalized the elevated expression of mGlu5 receptor in the hippocampus and prefrontal cortex in our study.

In addition, given the finding that combined mGlu5 receptor and mGlu2/3 receptor activity in the perirhinal cortex is important for recognition memory (Barker et al. 2006a, b), we also examined changes in expression of mGlu2 receptor in brain structures important for retrieval of recognition memory, such as the prefrontal cortex, the hippocampus, and the striatum. Although the magnitude of increase was similar between mGlu5 receptor and mGlu2 receptor in the hippocampus on abstinence day 2, the changes in mGlu2 receptor were not observed in the prefrontal cortex and striatum. Taken together, these data suggest that the mGlu5 receptor is more engaged in the effects of repeated ethanol exposure than the mGlu2 receptor. Moreover, the impact of ethanol on mGlu2 receptor is dependent on brain structure. Generally, in our experiments, changes in expression of mGlu5 receptor and mGlu2 receptor were the most evident in the hippocampus. This implies that the hippocampus plays an important role in ethanol-induced deficits in the retrieval of recognition memory during acute ethanol abstinence. Furthermore, VU-29 returned expression of mGlu2 receptor to the control in the hippocampus, as it did with mGlu5 receptor. Although, published data indicated that both mGlu5 and mGlu2 receptors in the hippocampus are independently involved in different forms of synaptic plasticity (Manahan-Vaughan 1997), we cannot rule out the possibility that functional linkage between these receptors exists in the hippocampus.

In summary, our experiments indicated that acute ethanol administration or withdrawal from repeated ethanol administration induced deficits in recognition memory that were associated with an increase in expressions of mGlu5 (hippocampus, prefrontal cortex, and striatum) and mGlu2 (hippocampus) receptor proteins in brain structures important for this type of memory. VU-29, a mGlu5 receptor-positive allosteric modulator, reversed/decreased ethanol-induced recognition memory deficits and normalized the expression of mGlu5 and mGlu2 receptors in the hippocampus and cortex. Thus, our data show the pivotal role of mGlu5 receptor in recognition memory impairment induced by acute and repeated ethanol administration. It also suggests the possible prospect for compounds acting as mGlu5 receptor-positive allosteric modulators in countering the effects of alcoholism.
